# Could chronic opioid use be an additional risk of hepatic damage in patients with previous liver diseases, and what is the role of microbiome?

**DOI:** 10.3389/fmicb.2024.1319897

**Published:** 2024-12-02

**Authors:** Giovanni Tarantino, Mauro Cataldi, Vincenzo Citro

**Affiliations:** ^1^Department of Clinical Medicine and Surgery, “Federico II” University Medical School of Naples, Naples, Italy; ^2^Section of Pharmacology, Department of Neuroscience, Reproductive Sciences and Dentistry, Federico II University of Naples, Naples, Italy; ^3^Department of General Medicine, “Umberto I” Hospital, Nocera Inferiore, Italy

**Keywords:** opioids, fentanyl, liver toxicity, gut flora dysbiosis, CYP 3A4, interleukins

## Abstract

**Summary:** Among illicit drugs, addiction from opioids and synthetic opioids is soaring in an unparalleled manner with its unacceptable amount of deaths. Apart from these extreme consequences, the liver toxicity is another important aspect that should be highlighted. Accordingly, the chronic use of these substances, of which fentanyl is the most frequently consumed, represents an additional risk of liver damage in patients with underlying chronic liver disease. These observations are drawn from various preclinical and clinical studies present in literature. Several downstream molecular events have been proposed, but recent pieces of research strengthen the hypothesis that dysbiosis of the gut microbiota is a solid mechanism inducing and worsening liver damage by both alcohol and illicit drugs. In this scenario, the gut flora modification ascribed to non-alcoholic fatty liver disease performs an additive role. Interestingly enough, HBV and HCV infections impact gut–liver axis. In the end, the authors tried to solicit the attention of operators on this major healthcare problem.

## Introduction

According to *Diagnostic and Statistical Manual of Mental Disorders, Fifth Edition (DSM-5),* drug abuse refers to the excessive use of sedatives/hypnotics/anxiolytics/stimulants, alcohol, caffeine, cannabis, hallucinogens, inhalants, and opioids. Both substance abuse and dependence were merged into the category of substance use disorders (SUDs) (American Psychiatric Association, 2013). Drug addiction involves the mesolimbic system. When rewarding stimuli are experienced, the dopaminergic mesolimbic system is activated leading to the release of dopamine from the targeted nuclei (Swanson, 2000; Baik, 2013). Contextually, the concentration of dopamine grows in the reward areas of the brain, evoking the so-called “stereotypical exhilaration and relaxation effects.” As a consequence, this dopamine flood in the reward pathway is distinctively associated with the addiction to the drug (Comer and Cahill, 2019). A recent survey on the adult population from European regions revealed that substance-attributable mortality rates were highest for tobacco smoking, followed by alcohol and illicit drugs (Peacock et al., 2018). Among illicit drugs, opioid abuse is skyrocketing in an unprecedented way with its unbearable burden of deaths (Wilson et al., 2020). Fentanyl is one of major contributors to both fatal and non-fatal overdoses in the United States. In this country, it is reported that in 2017, more than 70,237 fatalities were the result of drug overdoses. Of these, a high number such as approximately 50,000 were due to opioids, but coincidently over 20,000 of those deaths were caused by fentanyl alone (National Institute on Drug Abuse, 2023). Specifically, in the time interval 2017–2019, death rates involving synthetic opioids increased from 9.0 per 100,000 population to 9.9 in 2018 and accounted for an astonishingly 67.0% of opioid-involved deaths in 2018. The rates, quite similar among men and women, both aged ≥25 years, need to force healthcare system to set up comprehensive surveillance and prevention measures to try to reduce this high toll (Wilson et al., 2020). Fentanyl users exhibit a false sense of wellbeing, feelings of euphoria and relaxation, extreme happiness, quickly dissolving into difficulty thinking, speaking or walking, confusion, dizziness and coma. Without immediate treatment by trained professionals, death comes swiftly in minutes. The signs recognizing opioid overdose are small, constricted “pinpoint pupils,” falling asleep or losing consciousness, slow, weak or no breathing, choking or gurgling sounds, limp body, cold and/or clammy skin, and discolored lips and nails (Centre for Disease Control and prevention, 2023). Impaired liver function, evaluated by the means of Child-Pugh score, was identified as one of the most significant factors in determining variation in serum fentanyl concentrations (Kokubun et al., 2012). The last finding is primary to understanding that reduced metabolic processes could induce potential side effects of fentanyl, mainly when highly prevalent non-alcoholic fatty liver disease (NAFLD) and alcoholic liver disease (ALD) co-exist (Alkhouri et al., 2022), but not only.

## Aim of the review

This narrative review was organized with summarizing the history of research in this field and clarifying trends. It has been presented as a ‘conceptual frame’, providing a “rationale” for predictions about the relationships among variables, where the contents are separated according to various physio-pathological aspects. In this model, the central body is partitioned in sections, each composed of mechanistic events, which are discussed and evaluated. As core investigation, we reviewed evidence on the interference of NAFLD and/or ALD, without overlooking HBV and HCV infections, with opioid use/abuse. We intended to show that not only NAFLD and ALD may occur in the context of these drugs hepatotoxicity but also that these and other previous hepatic pathologies could influence the susceptibility to opioid hepatotoxicity. In this context, it may be useful to draw the attention of physicians and healthcare authorities on a possible, sometime severe, risk of liver damage subsequent to the chronic use of these substances, with fentanyl as a cornerstone, in patients with hidden or full-fledged ALD, NAFLD, or viral infections.

## Methods

To prepare this narrative review, the authors first interrogated PubMed,^1^ Scopus,^2^ and Embase^3^ to track recent evidence using the following keywords: Opioids addiction, opioids analogs, opiod overdose, opioid antagonist, opium contamination, substance use disorders, fentanyl pharmacokinetics, adverse drug events, nonalcoholic fatty liver disease, hepatic steatosis, gut-liver axis, gut microbiome, obesity, metabolic syndrome, alcoholic liver disease, hepatotoxicity, anesthetics toxicity, microsomal enzymes, liver enzymes, drug induced liver injury, microRNAs, animal models, prognostic factors, viral hepatitis, coronavirus-19 (revised on September 2024). As acronyms were used SUD, NAFLD, ALD, CYP450, COVID-19, ALT/AST, HCV, HBV, COVID-19, DILI, PKs, ADEs, and their combination, thesaurus system such as the Medical Subject Headings (MeSH) terms of the National Library of Medicine was referred to for selecting the appropriate keywords directly related to the topic of interest. Full tests and abstracts, when the previous ones were not available, were screened from January 2000 to April 2023, and successively on September 2024, after duplicates removed. Among the available published articles in English, both preclinical and clinical studies (case reports, epidemiological surveys, RCTs) were critically evaluated with particular attention on key results, limitations, suitability of the methods used to test the initial hypothesis, quality of the results obtained, interpretation of the results, and impact of the conclusions in the area. Additional references were identified in the list of references of previously retrieved articles. The exclusion criteria comprehended no methods described, scarce interpretation of the results, and content redundancy or gray literature. This search ended up in summing up a number of findings, mostly concerning inner mechanisms, linked to liver damage. The final step was citing and listing the researched references.

### Liver toxicity: from the beginning

The pioneering studies dealing with liver toxicity of fentanyl are based on its side effects as an anesthetic. Other potential hepatotoxicity can be inferred by the whole class of opioids with same therapeutical use. First of all, the terminology commonly adopted in this field of research should be clarified. Minimum alveolar concentration (MAC) is the concentration of a vapor in the alveoli of the lungs that is needed to prevent motor response to surgical stimulus (Minimum Alveolar Concentration, 2023). The 0.3 MAC indicates a comparable level of anesthetic potency, using the response of male Sprague–Dawley rats to tail clamping, and determining the 50% effective dose [ED50] value related to thiopental (Shingu et al., 1983).

A historical research on a single sub-anesthetic dose of fentanyl showed that it had no effect on liver enzymes. While rats receiving the same dose, i.e., 15.6 /mcg/kg bodyweight of fentanyl (0.3MAC), for 6 consecutive days showed increase of transaminases, mainly GPT, also known as ALT, after repeated injections of fentanyl, with no evidence of liver necrosis (Fassoulaki et al., 1986). The previous findings were not consistent with the results of another study using the hypoxic rat model. In fact, fentanyl caused the most severe hepatic injury with centrilobular localization when compared with several anesthetics, with exception of halothane (Fassoulaki et al., 1984).

Still, to make matters more complicated, all the anesthetics have long ago been hypothesized that could affect the liver through perturbing the permeability of liposome membranes and consequently impairing cell functions (Fassoulaki et al., 1984). Accordingly, it has been evidenced that enflurane, isoflurane, halothane, and fentanyl in an animal-based study were associated with the same degree of post-anesthetic hepatic “dysfunction,” which was judged minimal both in cirrhotic rats (if they were exposed by inhalation to carbon tetrachloride in air at weekly intervals for 12 weeks to induce cirrhosis) and in non-cirrhotic ones (Baden et al., 1985).

In eliciting hepatotoxicity, the role of the liver detoxification system is expected to be highlighted. Starting with the finding that isolated rat hepatocytes metabolize morphine to various compounds (morphinone-glutathione conjugate, normorphine, and morphinone), the addition of morphine to the isolated hepatocytes induced a marked decrease in the level of intracellular glutathione (GSH) and resulted in cell death. The formation of glutathione conjugate was correlated with the loss of GSH. The cytotoxicity of morphinone was higher than that of morphine (Nagamatsu et al., 1986). Following this line of research, a study showed that fatty livers (likely of alcoholic and non-alcoholic origin) have a weak compensation of hepatic GSH regulation, which fails under stress conditions, thus increasing the fatty liver’s susceptibility to oxidative damage (Grattagliano et al., 2003).

To confuse the issue whether opioids could induce liver necrosis, a very recent case report of a middle-aged man with a chronic history of drug abuse who presented, after an overdose of cocaine and heroin, with acute liver failure, renews interest in opioid hepatotoxicity (Dolkar et al., 2022).

It is necessary to stress that the kind of hepatic lesions evidenced in old articles focusing on the liver toxicity, extrapolating this datum on the basis of anesthetics, is different in severity, thus leading to uncertainties. Although there are several lines of evidence about a direct hepatic injury from opioids, ascertaining the presence/absence of hepatotoxicity of these drugs is rarely so simplistically cut and dried, and certainly research in this area is still in its infancy, while the topic is a matter of intense socio-political debate yet. The pieces of research dealing with hepatotoxicity by opioids are shown in Table 1.

**Table 1:** Findings from preclinical and clinical studies concerning the liver toxicity induced by opioids.

Authors	Results from the study	Reference number (year)
Kokubun et al.	Child-Pugh score determines variation in serum fentanyl concentrations, inducing potential side effects.	Kokubun et al. (2012)
Fassoulaki et al.	Findings of ↑ serum ALT after 6 days of 15.6 mcg/kg of fentanyl in male Sprague–Dawley rats with no liver necrosis	Fassoulaki et al. (1986)
Fassoulaki et al.	Fentanyl caused most severe hepatic injury with centrilobular localization in the hypoxic rat model	Fassoulaki et al. (1984)
Baden et al.	Fentanyl gave minimal hepatic dysfunction in both cirrhotic and non-cirrhotic rats like enflurane, isoflurane, and halothane.	Baden et al. (1985)
Nagamatsu et al.	The cytotoxicity of morphinone was higher than that of morphine due to marked ↓ levels of intracellular glutathione resulting in cell death.	Nagamatsu et al. (1986)
Dolkar et al.	A middle-aged man with a chronic history of drug abuse presented with acute liver failure after an overdose of cocaine.	Dolkar et al. (2022)

### Similar features of alcohol-induced liver damage and opioid cytotoxicity

Before pointing out the additional effects of contextual habits, i.e., heavy alcohol drinking and opioid use/abuse, the liver cytotoxicity by excessive ethanol consumption should be opportunely addressed. Interaction between CYP2E1, ethanol metabolites, and enhanced lipid peroxidation is linked to the pathogenesis of alcoholic liver disease. That said, even CYP2A6, CYP3A415 and.

CYP3A4 induction also leads to consequent generation of acetaldehyde and lipid peroxidation-derived protein-aldehyde adducts in the liver (Niemelä et al., 2000), which are hybrid compounds resulting by interaction of alcohol metabolites with other complex molecules, confirming previous findings in the liver of alcohol-treated rats (Niemelä et al., 1998). Moreover, when looking at the early phase of histological liver damage in patients abusing of alcohol beverages, the presence of protein adducts in the centrilobular region of the liver (the same one interested in opioid cytotoxicity) shows that “adducts” formation is one of the leading events in the molecular processes displayed during the ALD (Niemelä, 1999).

### Bidirectional relationship between alcohol/opioids and gut flora

Alcoholic and/or opioid addiction, separately and contextually, is modified by gut microbiome, in the sense that these substances, impacting on gut flora, alter drug biotransformation pathways, but it is also true the opposite. Morphine-induced microbial dysbiosis associated with the gut barrier disruption was completely reversed by transplanting placebo-treated microbiota into morphine-treated animals (Banerjee et al., 2016). Morphine metabolism and elimination, both of them, are dominant in assessing their efficacy and its adverse effects (Smith, 2009). The same is valid for alcohol toxicity, in that among the four main parameters, absorption, distribution, metabolism, and excretion only metabolism plays a central role. In fact, the elimination of ethanol from the body occurs primarily through metabolism (92–98% of dose) (Jones, 2019).

Indeed, that gut microbiota exerts an important role during enterohepatic circulation either before drug absorption or through various microbial enzymatic reactions in the gut, which is a cogent finding (Zhang et al., 2018). The importance of gut microbiota in providing information on drug metabolism is evidenced by *in vivo*, *in vitro*, *ex vivo*, *in silico,* and multi-omics approaches (Dhurjad et al., 2022).

### Gut–liver axis: the intersection between microbiome, liver metabolism, and inflammation

It is well-established that disruption of intestinal epithelial integrity bears as consequence the bacterial translocation from the gut (Schulzke et al., 2009). Indeed, both of these processes are mediated by toll-like receptor (TLR2 and TLR4) signaling in morphine-treated mice (Meng et al., 2013). Following the previous line of research, demonstrating that morphine treatment gives place to a primary Gram-positive bacterial dissemination, authors using a murine model of poly-microbial sepsis showed the induced, sustained upregulation of interleukin (IL)-17A and IL-6. The over-expression of IL-17A compromised intestinal epithelial barrier function, increased gut permeability, maintained bacterial dissemination, and elevated systemic inflammation. In keeping with previous findings, analysis of the gut microbiome showed that morphine treatment induced enrichment of the Firmicutes phylum mostly, and specifically the Gram-positive bacterial species *Staphylococcus sciuri*, *Staphylococcus cohnii*, and *Staphylococcus aureus,* as well as *Enterococcus durans*, *Enterococcus casseliflavus*, *Enterococcus faecium*, and *Enterococcus faecalis* in the gut microbiome. IL-17A neutralization protected barrier integrity and improved survival in morphine-treated animals (Meng et al., 2015). At this point, it is mandatory to report another gut dysbiosis induced by chronic alcoholic beverages. Alcohol is one of the main factors that alters the proper functioning of the gut, leading to a disruption of the intestinal barrier integrity that increases the permeability of the mucosa with a trend for a depletion of bacteria with anti-inflammatory activity, such as *Bacteroidetes* and *Firmicutes* phyla, and an increase in bacteria with pro-inflammation activity, such as *Proteobacteria* (Mutlu et al., 2012). Gut flora function, especially related to bile acid metabolism, can modulate alcohol-associated injury from the less to the more severe form, i.e., alcoholic cirrhosis (Bajaj, 2019). Microbiota changes might also alter brain function, and the gut–brain axis might be a potential target to reduce alcoholic relapse risk. Particularly, the expansion of *Bifidobacterium* and *Lactobacillus* suggests that probiotic interventions for patients with alcohol-related disorders could be useful (Dubinkina et al., 2017). It is salient to analyze another emerging aspect involving gut flora modifications and systemic inflammation, process not only linked to life styles (addiction and obesity) but also to the frailty of old people, who for chronic inflammatory diseases, such as rheumatoid arthritis or osteoarthritis, are on opioid therapy for persistent ache. Pain in the elderly population, burdened by physiological, pharmacological, and psychological aspects, is a major problem of caring in the geriatric setting. In a random chart review of 300 US veterans, 44% of those receiving an analgesic also received opioids (Chau et al., 2008). Opioids are one of the major causes of adverse drug events (ADEs) during hospitalization or shortly after discharge. Out of 10,917 patient records, 357 ADEs were identified, of which 28 (8%) involved opioids (Schutijser et al., 2020). Chronic low-grade inflammation has been speculated to accelerate the aging process, as well as frailty. Intestinal homeostasis employs a crucial role in healthy aging (Xu et al., 2021). In this sense, the prebiotic consumption modifies the intestinal microbiota but unfortunately has little action on markers of inflammation (Mysonhimer et al., 2023).

**Figure 1:**
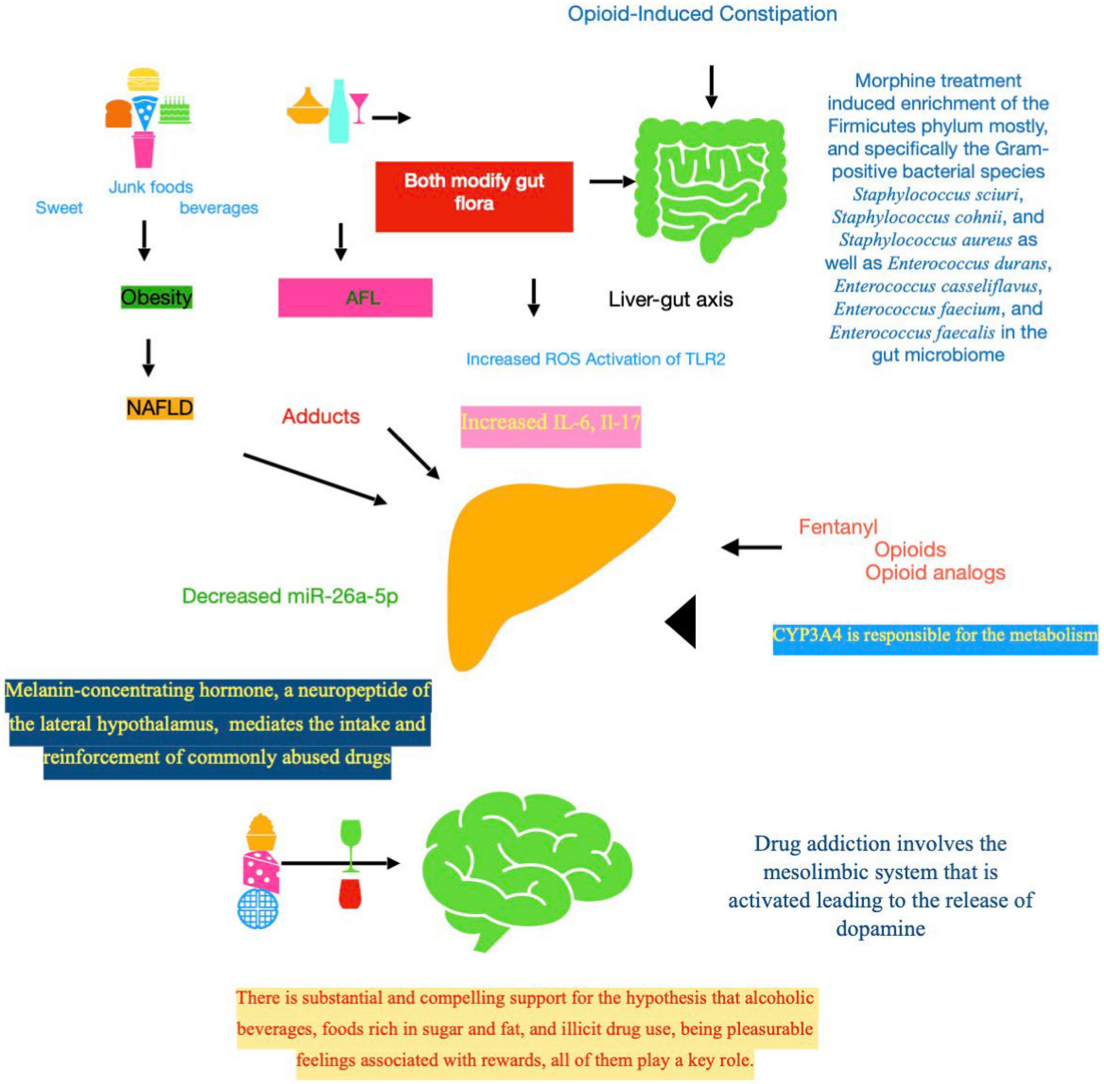
Downstream signaling events in opioid-related liver toxicity. First of all, it should be stressed that fentanyl contextually impacts on three levels, which are intertwined and bidirectional, i.e., CNS, gut flora, and liver. The complex ecosystem known as the gut microbiota, encompassing bacteria, viruses, and fungi living in the gastrointestinal system, plays a fundamental role in the control of the host energy metabolism. The liver–gut axis, mediated by intestinal microbiota, could be the link between co-existing liver disease, such as NAFLD and ALD, and the drug addiction. Acetaldehyde, a major toxic metabolite, is one of the principal culprits mediating fibrogenic of alcohol in the liver. Mechanistically, acetaldehyde promotes adduct formation, leading to functional impairments of key proteins, including enzymes. Indeed, gut microbiota of individuals with NAFLD produce enough ethanol to be a driving force in the development and progression of this disease. Gut microbial dysbioses are linked to aberrant immune responses, which are often accompanied by abnormal production of inflammatory cytokines (Il-6 and Il-17). The toxicity of opioids could be mediated also by *CYP3A4,* key enzyme involved in the fentanyl metabolism. MCH, a neuropeptide primarily transcribed in the lateral hypothalamus, displays multiple functions, mostly controlling feeding behavior and energy homeostasis and regulating the stress axis and emotion. MCH signaling pathway is a potential target to treat obesity, and also anxiety and depression that are at the basis of addiction. NAFLD, non-alcoholic fatty liver disease; ALD, alcoholic liver disease; ROS, reactive oxygen species; TRL, toll-like receptor; IL, interleukin; CYP, cytochrome P450 enzyme; SNC, central nervous system; MCH, melanin-concentrating hormone.

Returning to the intestinal microbiota, several lines of evidence suggest that obesity-related NAFLD could impact on patients who habitually use/abuse opioids. In a recent study, 72 cirrhotics chronically on opioids were age and severity—by model for end-stage liver disease and prior hepatic encephalopathy (HE)—balanced with 72 cirrhotics on no opioids. Stool microbiota composition (multi-tagged sequencing); predicted functionality, as estimated using so-called PiCRUST; endotoxemia; and inflammatory markers were comparatively evaluated. Significant gut flora modification was observed in the opioid cohort, especially in encephalopathic patients on opioids with lower abundance of the autochthonous families compared to others (Clostridiales XIV and *Lachnospiraceae*) along with a decrease in *Bacteroidaceae* relative abundance. PiCRUST showed the highest aromatic amino acid and endotoxin production in opioid users, who also had higher levels of IL-6. In contrast with previous findings addressing the intestinal permeability, in the aforementioned study, addicts were not characterized by increased levels of IL-17. Interestingly, the hypothesis of the authors was that opioids, being associated with obesity in that population, may predispose to the development or worsening of NAFLD. Surprisingly, alcoholic etiology did not impact on the gut flora (Acharya et al., 2017). The interference of opioids on cytokine profile is testified by the fundamental role of Il-6 in the development of morphine tolerance (Liu et al., 2019). The inflammatory cytokines, taken previously into account, engage a salient role also in patients with NAFLD, as possible liver co-morbidity of opioids dependents, mainly if the attention is focused on the microRNA-26a (miR-26a) that exhibits anti-inflammatory immune effects on immune cells (Yu et al., 2022). The mir-26a-IL-6-IL-17 axis attenuated NAFLD through inhibition of IL-6 in a murine model of high-fat diet-induced obesity. Moreover, IL-17 neutralization markedly decreased total liver weight, triglyceride deposition in liver (thus, hepatic steatosis), and serum transaminase (ALT) concentration when compared with the control group (He et al., 2017). Consequently, mir-26a, regulating insulin sensitivity and metabolism of glucose and lipids (Fu et al., 2015), was reduced in the livers of patients with NAFLD (Xu et al., 2021). Still, miR-26a-5p is hypothesized to carry out a protective role against DILI via targeting Bid, a pro-apoptotic member of the Bcl-2 family (Zhang et al., 2022). The reported findings have a certain significance in the light that neurovascular and immune system alterations are mediated by miRNA networks that function as regulators of cellular response to opioids (Grimm et al., 2023). Summing up, many molecular processes displayed, in the aforementioned studies, zero in both immunological and microbiological aspects. They are visible in Table 2 and Figure 1.

**Table 2:** Gut flora alterations associated with liver damage in course of opioid addiction and in presence of alcoholic and non-alcoholic fatty liver disease.

Authors	Findings from the study	Reference number (year)
Zhang et al.	Intestinal flora can influence pharmacokinetics, modulating therapeutic effects and side effects of drugs.	Zhang et al. (2018)
Meng et al.	Morphine induced in mice gut epithelial barrier dysfunction and subsequent bacteria translocation mediated by TLR2 and 4 signaling.	Meng et al. (2013)
Meng et al.	Morphine treatment induced enrichment of the Firmicutes phylum and specifically the Gram-positive bacterial species *Staphylococcus sciuri*, *Staphylococcus cohnii*, *Staphylococcus aureus, Enterococcus durans*, *Enterococcus casseliflavus*, *Enterococcus faecium*, and *Enterococcus faecalis* in the gut microbiome, using a murine model of poly-microbial sepsis due to activation of TLR2 induced sustained upregulation of IL-17A and IL-6.	Meng et al. (2015)
Mutlu et al.	Alcohol led to a disruption of the intestinal barrier integrity with ↑ permeability of the mucosa with depletion of bacteria with anti-inflammatory activity, such as *Bacteroidetes* and *Firmicutes* phyla, and ↑ in bacteria with pro-inflammation activity, such as *Proteobacteria* in 48 alcoholics.	Mutlu et al. (2012)
Bajaj et al.	Bile acid metabolism, related to gut flora, modulated alcohol-associated injury until alcoholic cirrhosis.	Bajaj et al. (2019)
Acharya et al.	Patients with encephalopathy+opioid use showed lower abundance of the autochthonous families Clostridiales XIV and *Lachnospiraceae* compared to others with ↓ in *Bacteroidaceae* relative abundance. PiCRUST evidenced highest aromatic amino acid and endotoxin production in opioid users, who had higher levels of IL-6.	Acharya et al. (2017)
Liu et al.	The interference of opioids on inflammatory cytokines is testified by the fundamental role of Il-6 in the development of morphine tolerance.	Liu et al. (2019)
Yu et al.	Interleukins exert a central role also in patients with NAFLD, mediated by microRNA-26a. exhibiting anti-inflammatory/immune effects.	Yu et al. (2022)
He et al.	The mir-26a-IL-6-IL-17 axis attenuated NAFLD through inhibition of IL-6 in a murine model of high-fat diet-induced obesity. IL-17 neutralization markedly decreased total liver weight, hepatic triglyceride deposition, and serum ALT concentration when compared with the control group.	He et al. (2017)
Xu et al.	Mir-26a, regulating insulin sensitivity and metabolism of glucose and lipids, was reduced in the livers of patients with NAFLD.	Xu et al. (2021)
Zhang et al.	MiR-26a-5p plays a protective role against DILI via targeting Bid, a pro-apoptotic member of the Bcl-2 family.	Zhang et al. (2022)
Grimm et al.	Neurovascular and immune system alterations are mediated by miRNA networks functioning as regulators of cellular response to opioid abuse.	Grimm et al. (2023)
Meijnikman et al.	There is an endogenous production of alcohol in NAFLD patients. *Lactobacillaceae* correlated with postprandial peripheral ethanol concentrations.	Meijnikman et al. (2022)

Continuing the focus on the impact of gut flora as foremost, but not unique, mechanism of liver damage, in addition to the role of glucose dysmetabolism (Bedogni et al., 2012), as well as the involvement of lipids (Pei et al., 2020) in the onset and progression of NAFLD, recently the endogenous production of alcohol is emerging. In fact, to test the hypothesis that the gut microbiota of patients with NAFLD produce enough ethanol to be a driving force in the development and evolution of this highly prevalent liver disease, the authors performed both a prospective and intervention clinical study, whose results suggested that microbial ethanol could be considered fundamental to the pathogenesis of NAFLD. Furthermore, *lactobacillaceae* correlated with postprandial peripheral ethanol concentrations (Meijnikman et al., 2022). This last finding apparently closes the circle. In conclusion, based on the above studies, there is likely an association between the intestinal microbiota and liver disease, but a causal relationship has yet to be confirmed (Schwenger et al., 2019).

Coming back to metabolic dysfunctions in opioid addicts, not always overlapping, as evident in Table 3, a systematic review comprehending 46 articles and 37,407 participants suggested that opioids increase serum triglycerides (thus, inducing or worsening hepatic steatosis) and may reduce blood glucose and low-density lipoproteins (LDLs). However, these effects are temporary, even though long-term drug dependence exacerbates glucose and lipid-associated diseases such as diabetes mellitus and atherosclerosis (Rezaei and Rezaei, 2021). In keeping with precedent data on LDL, the authors of a recent meta-analysis showed that opium abuse significantly decreased total cholesterol compared to that of controls but only in diabetic patients. On the contrary, concerning body mass index (BMI), the differences were not statistically significant. Those researchers concluded that nutritional deficiencies, weight loss, and lipid dysregulation due to liver dysfunction (as sequela of previous diseases) may explain the findings (Ojo et al., 2019). On the other side, compelling results show that chronic heroin administration produces a state of fasting hyperinsulinemia (again, another driver of NAFLD) even in the absence of obesity (Passariello et al., 1983). Further data show that alteration of glucose metabolism evidences similarities between opiate addicts and non-insulin dependent diabetics (Ceriello et al., 1987). Indeed, the lack of involvement of BMI in opioid abusers is challenged by other pieces of research. Starting with the growing consensus that the overconsumption of readily available and highly palatable foods likely contributes to the growing rates of obesity worldwide (Johnson and Wardle, 2014), the high intake of ultra-processed foods was associated with an increased risk of NAFLD, as evidenced in 6,545 participants who were recruited in National Health and Nutrition Examination Surveys 2011–2018 (Liu et al., 2022), but the point is that both preference and consumption of sweet substances often parallel the self-administration of several drugs of abuse, and under certain conditions, the termination of intermittent access to sweet substances produces symptoms that resemble those observed during opiate withdrawal (Gosnell and Levine, 2009).

**Table 3:** Metabolic dysfunctions in opioid addicts.

Authors	Findings from the study	Reference number (year)
Rezaei et al.	Systematic review showing that opioids ↑ serum triglycerides and ↓ blood glucose and low-density lipoproteins.	Rezae and Rezaei (2021)
Ojo et al.	Meta-analysis showing that levels of total cholesterol were ↓ in diabetic patients, abusing of opium. Nutritional deficiencies, weight loss, and lipid dysregulation, due to liver dysfunction, may explain the findings.	Ojo et al. (2019)
Passariello et al.	Chronic heroin administration produced a state of fasting hyperinsulinemia even in the absence of obesity.	Passariello et al. (1983)
Passariello et al.	Alteration of glucose metabolism evidenced similarities between opiate addicts and non-insulin dependent diabetics.	Ceriello et al. (1987)
Gosnell et al.	Preference/consumption of sweet substances often parallels the self-administration of several drugs of abuse, and the termination of intermittent access to sweet substances produces symptoms that resemble those observed during opiate withdrawal.	Gosnell and Levine, (2009)
Zahmatkesh et al.	Opioids ↑ the levels of ROS and ↓ those of the activity of superoxide dismutase, catalase, and glutathione peroxidase that function as enzymatic antioxidants. Furthermore, they ↑ the risk of vitamin deficiency and modify gene expression of target cells through ROS production.	Zahmatkesh et al. (2017)
Nabipour et al.	Many opiate and alcohol addicts present with ↓ Ca and ↓ Mg due to poor diet and inadequate intake of *Ca.*	Nabipour et al. (2014)
Skrabalova et al.	Production of ROS/nitrogen species can contribute to degenerative diseases and organ dysfunction, likely of liver, occurring in morphine abusers or morphine-treated patients.	Skrabalova et al. (2013)
Reece et al.	1,602 patients with SUD compared with 2,858 non-SUD patients showed an acceleration of age-related and degenerative pathologies in many tissues, evaluated by the higher levels of serum glucose, HbA1c elevation, fructosamine, concentrations, and microalbumin levels.	Reece et al. (2013)
Duailibi et al.	A systematic review of the RCTs present into literature until 2017 showed the safety profile of N-acetylcysteine and its favorable tolerability, in addition to being an over-the-counter medication with an interesting potential clinical use for craving in SUDs.	Duailibi et al. (2017)
Lewinska et al.	Paradoxically, researchers have found that a preconditioning of one potent, short-acting synthetic opioid analgesic drug that is remifentanil could protect against hypoxia-induced cellular senescence.	Lewinska et al. (2020)
Dimidi et al.	Playing gastrointestinal microbiota a primary role in constipation, modification of the gut luminal environment, with certain probiotic ends up in improvement of the motility and secretion in the gut, resulting in *bifidobacteria* and *lactobacilli.*	Dimidi et al. (2017)
Jang et al.	A link between constipation from opioids and the underlying NAFLD is hypothesized by the fact that *Lactobacillus* decreases intestinal lipid absorption, consequently protecting against diet-induced steatosis *in vivo.*	Jang et al. (2019)
Mallappallil et al.	Opioid use can lead to a reduced fluid intake.	Mallappallil et al. (2017)
Van Vugt et al.	Administration of opiates produces a rapid increase in release of ADH.	Van Vugt and Meites (1980)

Possible interactions between opioid abuse and organ senescence bring us to other pieces of research dealing with impaired oxide reductive processes, mostly involved in drug addicts, as well as in patients suffering from ALD and NAFLD. Cellular senescence is stress-responsive program limiting the proliferation of damaged cells and leading to stable cell cycle arrest. An increase in ROS levels has been demonstrated to be crucial to inducing and maintaining senescence process (Davalli et al., 2016). Hepatocyte senescence can represent a foremost mechanism inducing intracellular fat accumulation, fibrosis and inflammation, characteristics of NAFLD, and its more severe form, i.e., non-alcoholic steatohepatitis, due to secretion of senescence-associated inflammatory mediators (Engelmann and Tacke, 2022). Analyzing sub-sequential molecular events related to the injury toward hepatocytes, alcohol stimulates the activity of cytochrome P450 enzymes, which contribute to ROS production, and reduces the levels of the antioxidant system (Wu and Cederbaum, 2003). Consequently, ethanol-induced oxidative stress causes DNA damage and defective DNA repair, inhibiting a main DNA repair factor that is 53BP1. The improperly repaired DNA damage further activates cell cycle checkpoint proteins, such as p53 and p16INK4α, and leads up to the onset of premature senescence (Romero et al., 2016). Finally, senescence markers, such as the transcription factors E2F1 and ID1 and the insulin-like growth factor binding protein-3, were significantly altered in ethanol-fed mouse liver specimens compared to controls (Meng et al., 2017). On the other hand, opioids increase the levels of ROS and decrease the activity of superoxide dismutase, catalase, and glutathione peroxidase that function as enzymatic antioxidants. Moreover, opioids augment the risk of vitamin deficiency and modify gene expression of target cells through ROS production (Zahmatkesh et al., 2017). To make matter worse, many opiate and alcohol addicts present with calcium and magnesium deficiencies due to poor diet and inadequate intake of calcium (Nabipour et al., 2014). Recently, there are many literature data indicating a key involvement of calcium signaling in cellular senescence (Martin and Bernard, 2018), as well as of magnesium (Killilea and Maier, 2008). Conclusively, production of ROS/nitrogen species can contribute to degenerative diseases and organ dysfunction, likely also of liver, occurring in morphine addicted or morphine-treated patient (Skrabalova et al., 2013). To prove the importance of these biochemical pathways, a recent research enrolling 1,602 patients with SUD compared with 2,858 non-SUD patients showed that there is an acceleration of age-related and degenerative pathologies in many tissues, evaluated by the higher levels of serum glucose, HbA1c elevation, fructosamine, concentrations, and microalbumin levels, all of them being biomarkers of age (Reece, 2013). In line with the centrality of oxidation–reduction process involved in drug abuse, a systematic review of the RCTs present into literature until 2017 showed the safety profile of N-acetylcysteine and its favorable tolerability, in addition to being an over-the-counter medication with an interesting potential clinical use for craving in SUDs (Duailibi et al., 2017). This is to cast a fresh light on therapeutical approach to SUD. Paradoxically, researchers have found that a preconditioning of one potent, short-acting synthetic opioid analgesic drug that is remifentanil could protect against hypoxia-induced cellular senescence (Lewinska et al., 2020).

### How does constipation contribute to opioid toxicity and what is the link between opioid-induced constipation and infections?

Now, it is required to deepen how other deleterious consequences of opioid abuse can modify the hepatocyte integrity adding further damage to the preexisting alcohol or metabolic one. That opioids have a poor side effect profile which is ascertained. Indeed, the incidence of opioid-induced constipation is consistently high, varying from 15 to 81% (Lang-Illievich and Bornemann-Cimenti, 2019). Administration of opiates produces a rapid increase in release of ADH, (Van Vugt and Meites, 1980), as well as opioid use can lead to a reduction of fluid intake (Mallappallil et al., 2017). Fluid restriction increases constipation (Arnaud, 2003). Opioid-induced bowel dysfunction, manifesting as constipation, is mediated by peripheral *μ*-opioid receptors with resultant altered gastrointestinal motility (Lee and Hasler, 2016). Among factors impacting on gut motility, and thus constipation, beyond the action of immune and nervous system, the metabolism of bile acids, and secretion of mucus, the gastrointestinal microbiota is relevant. In fact, modifying the gut luminal environment, i.e., decreased numbers of *bifidobacteria* and *lactobacilli* in stool sample, with some probiotics, the motility and secretion in the gut are improved (Dimidi et al., 2017). To try to find a link between constipation from opioids and the possible underlying NAFLD of these subjects, recent observations hypothesize that *Lactobacillus* decreases intestinal lipid absorption, consequently protecting against diet-induced steatosis *in vivo* (Jang et al., 2019). Researchers have seen that opioids can lead to a dehydration status (Mallappallil et al., 2017), but it should not be overlooked the fact that also ethanol ingestion affects the hypothalamo-neurohypophysial system resulting in increased diuresis, dehydration, and hyperosmolality, thus summing up the effects of dehydration to altered microbiome (Madeira et al., 1993). Accordingly, recent pieces of research indicate that drinking water may be an important factor in shaping the human intestinal flora (Vanhaecke et al., 2022). In fact, euhydration and dehydration states determine changes in microbiota community and the immune response (Lukito, 2021). To get a further glimpse on microbiota, it is necessary to highlight that many infections of immunocompromised patients, such as alcohol addicts (Szabo and Saha, 2015), originate from the gastrointestinal tract, with alteration of the whole flora (Taur and Pamer, 2013). In fact, bloodstream infections are a frequently observed complication in liver cirrhosis patients of alcoholic origin (Xie et al., 2017). Stressing that concentrations of *Bifidobacterium* and *Lactobacillus* are significantly lower in constipated patients, potentially pathogenic bacteria and/or fungi could be increased (Khalif et al., 2005). In fact, the link between opioid-induced constipation and infection is represented by the frequent association of constipation with urinary tract infection (UTI) and upper urinary tract dilatation (Naseri et al., 2022). That gastrointestinal tract is one of the main anatomical regions implicated in the pathophysiology of UTIs, which is proved by the finding that intestinal dysbiosis may upregulate the expression of serotonin transporter involved in regulating gastrointestinal motility and contribute to the development of chronic constipation, supporting a never-ending vicious circle (Cao et al., 2017). To support the connection between drug abuse and constipation, intriguingly, opioid use disorder may lead to the development of primary hypothyroidism (Pezeshki et al., 2023), disease in which constipation is frequently observed (Pustorino et al., 2004). Coming back to infections, it should be highlighted that fentanyl fundamentally exerts a relevant immunosuppression (Sacerdote, 2008). Because each functional compartment of the immune system plays a specialized role in host defense, defects in specific functions lead to increased susceptibility to specific pathogens (Dropulic and Lederman, 2016). In addition, use of both opioids and other substances through IV administration places individuals at increased risks of infectious diseases ranging from invasive bacterial and fungal infections to human immunodeficiency virus and viral hepatitis (Marks et al., 2022). In this context, it has been evidenced how fungal products (beta-glucans and candidalysin) activate the host’s immune system to exacerbate liver and biliary diseases (Hartmann and Schnabl, 2023).

### The role of cytochrome P450 enzymes

**Figure 2:**
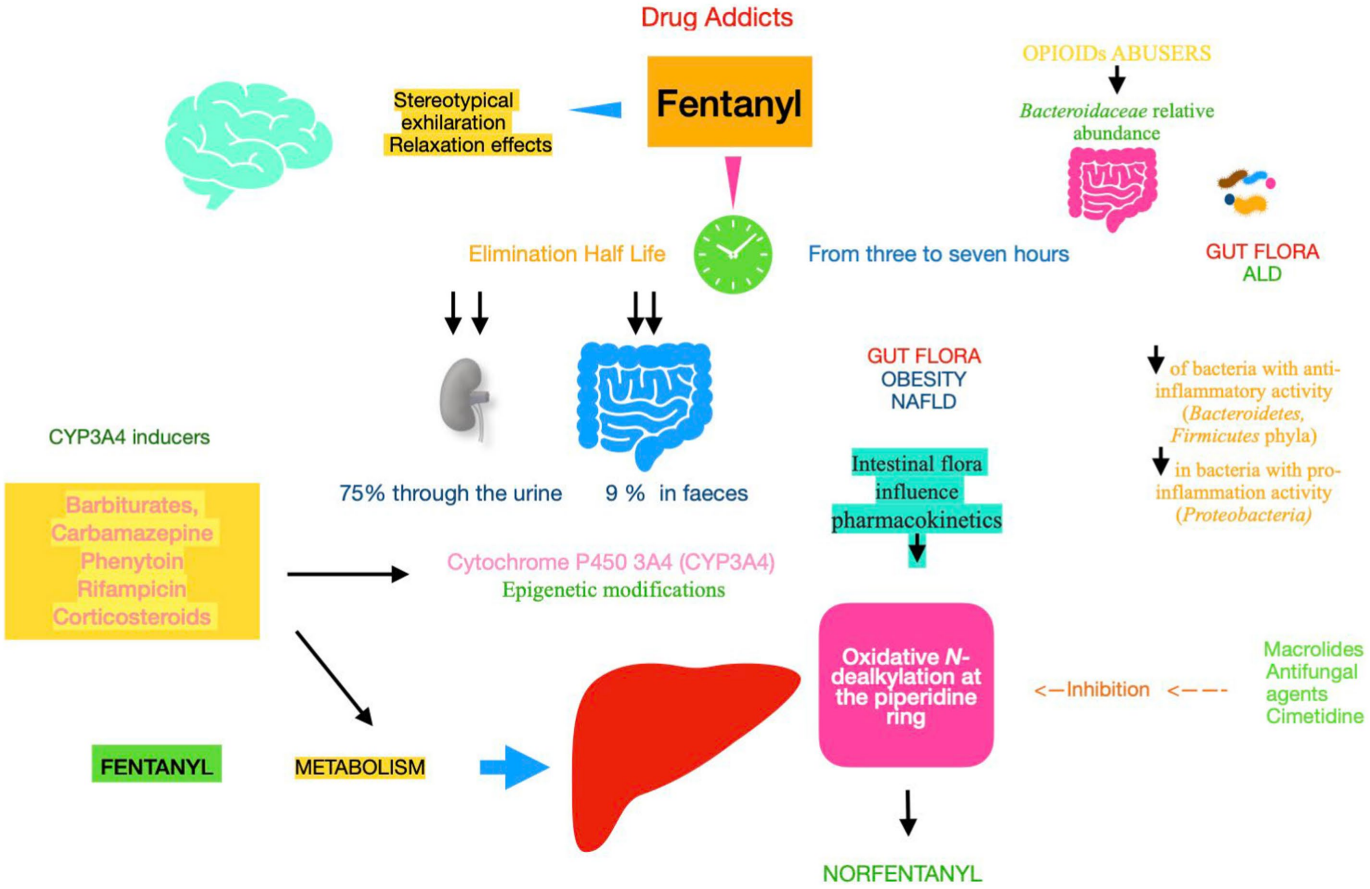
Pharmacokinetics and metabolism of fentanyl Fentanyl metabolism and elimination (half-life), both of them, play an important role in determining drug pharmacokinetics and assessing their efficacy and its adverse effects. Gut microbiota plays a key role during enterohepatic circulation along the drug absorption phase and mainly through various microbial enzymatic reactions in the gut. Fentanyl is 99% N-dealkylated to norfentanyl by cytochrome P450. Utilizing a proteomics approach to quantify the protein expression of CYP3A4 and related enzymes, the results suggest that NAFLD is associated with the decreased hepatic CYP3A4 activity. Epigenetic changes in CYP genes would lead to inter-individual differences in drug responses. Understanding epigenetic mechanisms can help reduce toxicity for drugs metabolized by CYP enzymes. NAFLD, non-alcoholic fatty liver disease; ALD, alcoholic liver disease; CYP, cytochrome P450 enzyme.

To give a broader view of the mechanisms underlying the possible liver damage, the metabolic pathways of opioids should be accentuated. Specifically, could liver microsomes exercise a significant part in inducing opioid hepatotoxicity, independently from the absorption and excretion of drugs? This has naturally led to deep dispute within the field. In human liver microsomes, there were no statistically significant differences in CYP activity as a function of age, gender, or ethnicity with *CYP3A4* activity being slightly greater in women than men (Parkinson et al., 2004). Utilizing a proteomics approach to quantify the protein expression of *CYP3A4*, the results suggest that NAFLD and diabetes mellitus are associated with the decreased hepatic *CYP3A4* activity (Jamwal et al., 2018). In keeping with the previous findings, recent results demonstrate that the transcription repression of the *CYP3A4* activity in turn promotes liver fat excess (Huang et al., 2019), thus worsening NAFLD. Consequently, changes in definite cytochrome P450 enzymes, as those previously evidenced in livers of patients with steatosis, but also *in vivo* models of steatosis (in experimental animals) and *in vitro* models of fat-overloaded cells, pose major impact on drug-induced hepatotoxicity (Gómez-Lechón et al., 2009), on the basis that *CYP 3A4* is the main responsible for the metabolism of fentanyl (Figure 2) (Tateishi et al., 1996). Among the various factors that may influence the pharmacological response to opioids, genetic polymorphisms have generated some interest. A recent meta-analysis including 59 studies showed that CYP3A4*1G carriers consumed less opioids than homozygous CYP3A4*1/*1 patients during the first 24 h of the postoperative course (Ren et al., 2015). Using a preclinical model, the authors evidenced that chronically fentanyl-treated animals (male Brown Norway X F344 rats aged 12, 18, 24, and 30 months) lost both fat and lean mass during early and late drug administration, as well as during early withdrawal, independently from aging (Mitzelfelt et al., 2011). To evaluate the link between *CYP3A4* polymorphisms and drug addiction risk, the authors found that in the Chinese Han population, rs4646440 and rs4646437 were associated with decreased risk of drug addiction in co-dominant and recessive models, while rs3735451 and rs4646437 were associated with drug addiction risk in the middle-aged and elderly people. Finally, rs3735451, rs4646440, and rs4646437 had strong relationship with decreased risk of drug addiction for men (Wang et al., 2019). Considering the high inter-subject variability of CYP 450, the activity of this superfamily of enzymes has been largely attributed to gene polymorphism. Indeed, this interpretation has quite completely changed following the rapid development in epigenetics that has revealed another aspect of regulatory mechanism of drug-related genes (Tang and Chen, 2015). Among the many epigenetic factors, affecting CYP expression, beyond histone modification, non-coding (nc)RNA regulation was particularly deepened. Of the ncRNA classes, miRNAs play a key role (Nakano and Nakajima, 2018). Numerous “stressors” from various sources, including food and alcohol (again the link with liver diseases), tobacco, air pollution, some pharmaceuticals, and commercial products, have been shown to alter the expression of miRNAs (Marrone et al., 2014). Furthermore, exposure to DNA methylating agents may lead to hyper-methylation of CYPs genes by DNA methyltransferases and inhibition of CYP expression. Consequently, also DNA methylation potentially contributes to the inter-individual expression of *CYP3A4 i*n drug metabolism and associated adverse drug effects (Kacevska et al., 2012). It is essential to underline that chronic alcohol consumption, the cause of ALD, leads to enhanced activity of another member of the CYP family, i.e., *CYP2E1*, in the smooth endoplasmic reticulum (Leung and Nieto, 2013). Consequently, this difference could have potential major implications for the risk of drug toxicity.

### Will opioid use contribute to liver damage within the context of the viral infection?

New cases of acute hepatitis C virus (HCV) have increased rapidly in the US since 2010 and have most often been associated with injection drug use. Similarly, a recent national boost in acute hepatitis B (HBV) has coincided with an epidemic of opioid abuse (Centers for Disease Control and Prevention, 2017). As a consequence, there is a significant rise in the proportion of organ donors who died due to overdose, but a better use of donors in the light of the risk of disease transmission is needed (Verna et al., 2019). However, there is evidence that opioid agonist therapy is associated with increased odds of HCV treatment initiation among people who use drugs (Bartlett et al., 2022). Exogenous opioids, such as morphine and fentanyl, have been found to impair the function of macrophages, natural killer cells, and T cells and weaken the gut barrier *in vitro* and in animal studies (Plein and Rittner, 2018). The opioid-induced immunosuppression, coupled with the release of cytokines, hormones, and bacterial products from intestinal tract, could explain from a hand the lack/slowness of viral clearance and on the other hand the ongoing liver injury. Drug users who are successfully treated for HCV infection can become reinfected if they inject unsafely (Proust et al., 2000). That said, fentanyl kinetics, studied in patients with cirrhosis, showed that its elimination half-life is not primarily influenced by the rate at which it is metabolized in the liver (Haberer et al., 1982). Surprisingly, HCV infection impacts gut–liver axis by altering the composition of gut microbiota (dysbiosis), characterized by a loss of microbial diversity and the expansion of potential pathogens (Marascio et al., 2022).

Furthermore, recent findings provide evidence of alterations of the gut microbiome and some related metabolites in patients with both immunotolerant or immuno-active phase of HBV infection (Li et al., 2022). By these findings, it is likely that the aforementioned diseases could impact on pharmacokinetics of opioids.

Concerning another viral infection, in one cross-sectional study, deaths due to opioid toxicity increased substantially during the COVID-19 pandemic (Mamdani et al., 2023). Intriguingly, although most patients with COVID-19 have a mild increase in transaminases, the highest rate of liver damage is in adult patients (Sadeghi Dousari et al., 2023). Obviously, the liver damage induced by this viral infection could be worsened by a contextual drug toxicity, mainly in patients with long-term COVID-19, whose laboratory data show ALT and AST levels persistently elevated (de Lima et al., 2023). An important point is that patients on opioid overdosing can present rhabdomyolysis but are often not diagnosed (Babak et al., 2017). Rhabdomyolysis could mimic liver damage, presenting with a marked transaminases elevation (Lim, 2020), and causes acute kidney injury (Hebert et al., 2022). Finally, also fentanyl overdose creates the same renal damage (Mallappallil et al., 2017).

## Discussion

Surfing the literature, the answer to the initial question whether there is evidence of liver toxicity by opioids, synthetic opioids and mainly by fentanyl is positive. However, many aspects remain unsolved with an unmet need to deepen the topic, in relation to the ways in inducing liver damage, and the timing of use/abuse of these drugs, theme that is subject of much controversy, i.e., pain control. Our main aim, addressing an important question in the light of skyrocketing increase of obesity and alcohol addiction, especially among youngsters, was digging into the results of some studies to draw the evidence that the co-presence of NAFLD and ALF, likely mediated by gut flora dysbiosis, could impact on the onset and progression of these very widespread diseases. Placing this review within the context of previous studies, the authors ought to report that data from literature emphasize the main role of gut microbiome in all the three pathologies, such as opioid addiction and both NAFLD and ALD, suggesting that there could be an additive effect in inducing hepatic injury. First, it is intriguing to look at how the results of the selected studies could be important to the audience. The study of the intestinal microbiota reveals that it is constantly evolving in response to host as age, nutrition (the role of rich-calorie foods in obesity), lifestyle (alcoholic beverage abuse), hormonal changes, inherited genes, epigenetic modifications, and underlying disease (NAFLD and ALD as possible examples) are major determinants of the human microbiome at any given point in time (Ogunrinola et al., 2020). As correct hypothesis, a balanced microbiota has a fundamental role in health maintenance. However, gut dysbiosis is a key aspect in linking prior and, in most cases, evolving hepatic diseases with opioid addiction. In fact, alterations in the intestinal microbiota can impact drug metabolism, favoring more toxic metabolites, ultimately injuring liver tissues (hepatotoxicity), and in turn worsening the preexisting alcohol or metabolic liver damage. On the other hand, altered drug metabolism enzyme expression has been found in rodent and human samples of NAFLD (Zhu et al., 2023), with the result that underlying metabolic-induced damage could give place to or worsen liver toxicity by opioids. Nevertheless, without lessening the importance of gut flora, the role of microsomal enzymes, specifically CYP 450, should not overlooked, mainly concerning the genetic expression and epigenetic modifications, as presented in interesting studies. Summing up the aforementioned evidence concerning the opioid-related liver toxicity, there is substantial and compelling support for the hypothesis that alcoholic beverages, foods rich in sugar and fat, and illicit drug use, being pleasurable feelings associated with rewards, all of them conduct a determinant contribution. Research shows that there is a link between substance abuse and obesity, as well as obesity-associated diseases, in brain functioning. In keeping with this evidence, current literature emphasizes the role of melanin-concentrating hormone (MCH), a neuropeptide primarily transcribed in the lateral hypothalamus, in mediating the intake and reinforcement of commonly abused drugs (Morganstern et al., 2020). By the same accord, the opioid antagonist naltrexone blocks the endogenous opioid-mediated inhibition of anorexigenic pro-opiomelanocortin (POMC) neurons, leading to sustained POMC stimulation, thus acting on central appetite and reward region (Walmsley and Sumithran, 2023). Fascinating findings evidence that central MCH directly controls hepatic and adipocyte metabolism through different pathways (Imbernon et al., 2013). Furthermore, intracerebroventricular infusion of MCH caused induction of hepatic steatosis and increase in body weight (prominent aspect) in high-fat diet-fed wild-type mice (Kawata et al., 2017). It is emerging that alcohol excess, apart its direct hepatotoxicity, is of paramount importance in leading to weight gain, mainly when associated with illicit drug dependence and negative eating behaviors especially in young and very young people (Tarantino et al., 2022). The high prevalence of NAFLD all over the world is associated with the pandemic of obesity. This explosion is testified by an updated analysis finding that mean BMI increased from 23.0 (95% CI, 22.8–23.2) in 1976–1980 to 27.5 (95% CI, 25.5–29.4) in 2017–2018, and the prevalence of obesity increased from 5.5% (95% CI, 4.3–7.0%) to 32.6% (95% CI, 22.1–45.2%) (Ellison-Barnes et al., 2021). In this scenario, the gut flora modification ascribed to NAFLD develops an additive role (Hrncir et al., 2021). To reinforce the pivotal role of microbiota, similar downstream signaling events are involved in the onset and progression of ALD and NAFLD (Fleming et al., 2020). A large proportion of street-purchased opioids includes fentanyl, and the drug has also become an adulterant within the stimulant supply (Tarantino and Citro, 2024). To stem this tide, also the hepatologists’ awareness is imperative to increase, coupled with other specialists, beyond educational programs (Office of Addiction Services and Supports, 2023). Limitations to this review consist in the paucity of specific data of literature concerning the models of liver toxicity by opioids that does not permit deepening the sub-sequential molecular processes displayed by the use/abuse of these drugs. Still, a drawback is the lack of clinical trials, utilizing robust methodologies and advanced statistical analysis, in patients contextually suffering from NAFLD and drug abuse, as well as studies addressing both alcohol and drug dependence, focusing on the possible liver damage. It needs to be emphasized, as final observation, that authors do not know for sure whether the modifications of the microbiota are cause, effect, or side effect of opioid addiction in the context of other hepatic diseases.

### Future directions

The authors would suggest some other field of research, starting from studies documenting that endogenous and exogenous opioids not only induce analgesic effects by regulating pre- and post-synaptic sensory neurons but also interact with opioid receptors present in the immune system, resulting in immune modulation (Ninković and Roy, 2013). Specifically, the release and diffusion of neurotransmitters favor signaling through lymphocyte cell surface receptors, which regulates the immune response (Sacerdote et al., 2008). Interesting findings suggest that the immune system abnormalities in heroin addicted patients can be restored to almost normal values by controlled treatment with methadone and buprenorphine (Oshaghi et al., 2023). Fascinating enough, opioids can favorably harmonize the neurotransmitter systems controlling mood and are suggested in the treatment of psychiatric disorders (Kawata et al., 2017). Finally, the wide spreading use of antibiotics (English and Gaur, 2010) has serious effects on the host through the gut microbiome and can affect various functions including immune regulation, metabolic activities, and thus overall health (Patangia et al., 2022). It would be captivating to establish whether antibiotic-disrupted gut flora contribute to liver toxicity by opioids and whether phage therapy could be proposed as a clinical alternative to restore intestinal eubiosis, due to its immunomodulatory and bactericidal effect against bacterial pathogens (Gutiérrez and Domingo-Calap, 2020), and consequently reduce liver damage in these addicts. Basing on the finding that magnesium reduces the intensity of addiction to opioids (Magnesium in the Central Nervous Systems, 2023), another interesting approach could be evaluating the use of this essential mineral in trying to alleviate the morphine-induced tolerance and withdrawal symptoms in humans, as was found in mice with administration of lamotrigine or magnesium sulfate or their combination (Habibi-Asl et al., 2009). Still, the main role of the epigenetic changes in CYP genes, leading to inter-individual differences in drug responses, should be stressed to decrease adverse drug reactions for drugs metabolized by CYP enzymes. Specifically, new studies are needed to develop CYP-based pharmacoepigenetics to guide clinical applications for precision medicine with reduced risk of toxicity (Jin and Zhong, 2023). As significant subject, extracellular vesicles may start providing information about mechanisms and pathogenesis in substance use disorders, consenting to potential therapeutic options being probed (Odegaard et al., 2020). Finally, to raise awareness of this nationwide epidemic, educational presentations for students in all age ranges, from elementary school through graduate school, are supposed to be implemented.

## Conclusion

The “take-home” message that the authors want our readers to leave with is that there is proof of a close link between key topics such as gut microbiome, liver metabolism, and inflammation in hepatic toxicity in opioid addicts. On this basis, the preexisting liver diseases, such as ALD or NAFLD, could worsen liver toxicity from opioid abuse, as well as opioid consume could bring further damage to the aforementioned liver diseases with complex and difficult to fully address mechanisms. Finally, the interdisciplinary nature of the topic, linking addiction medicine, hepatology, and microbiology, also makes it an interesting and relevant area of study to promote the initial and deserving interest of scientists toward this issue, unfortunately lost in time.

## Footnotes

^1^https://pubmed.ncbi.nlm.nih.gov/, accessed on April 2023

^2^https://www.scopus.com/search/form.uri?display=basic&zone=header&origin=#basic, accessed on April 2023

^3^https://www.embase.com/, accessed on April 2023
